# The Use of 3D Printed Vasculature for Simulation-based Medical Education Within Interventional Radiology

**DOI:** 10.7759/cureus.4381

**Published:** 2019-04-03

**Authors:** Christine Goudie, Jason Kinnin, Michael Bartellas, Ravindra Gullipalli, Adam Dubrowski

**Affiliations:** 1 Medical Education and Simulation, Memorial University of Newfoundland, St. John's, CAN; 2 Radiology, University of Saskatchewan College of Medicine, Saskatoon, USA; 3 Otolaryngology, University of Ottawa, Ottawa, CAN; 4 Radiology, Memorial University of Newfoundland, St. John's, CAN; 5 Emergency Medicine, Memorial University of Newfoundland, St. John's, CAN

**Keywords:** simulation, vasculature, 3d printing, medical education, radiology, interventional radiology, simulation based medical education, point of care, health care

## Abstract

Three-dimensional (3D) printing has become a useful tool within the field of medicine as a way to produce custom anatomical models for teaching, surgical planning, and patient education. This technology is quickly becoming a key component in simulation-based medical education (SBME) to teach hands-on spatial perception and tactile feedback. Within fields such as interventional radiology (IR), this approach to SBME is also thought to be an ideal instructional method, providing an accurate and economical means to study human anatomy and vasculature. Such anatomical details can be extracted from patient-specific and anonymized CT or MRI scans for the purpose of teaching or analyzing patient-specific anatomy. There is evidence that 3D printing in IR can also optimize procedural training, so learners can rehearse procedures under fluoroscopy while receiving immediate supervisory feedback. Such training advancements in IR hold the potential to reduce procedural operating time, thus reducing the amount of time a patient is exposed to radiation and anaesthetia.

Using a program evaluation approach, the purpose of this technical report is to describe the development and application of 3D-printed vasculature models within a radiology interest group to determine their efficacy as supplementary learning tools to traditional, lecture-based teaching. The study involved 30 medical students of varying years in their education, involved in the interest group at Memorial University of Newfoundland (MUN). The session was one hour in length and began with a Powerpoint presentation demonstrating the insertion of guide wires and stents using 3D-printed vasculature models. Participants had the opportunity to use the models to attempt several procedures demonstrated during the lecture. These attempts were supervised by an educational expert/facilitator.

A survey was completed by all 30 undergraduate medical students and returned to the facilitators, who compiled the quantitative data to evaluate the efficacy of the 3D-printed models as an adjunct to the traditional didactic teaching within IR. The majority of feedback was positive, supporting the use of 3D=printed vasculature as an additional tactile training method for medical students within an IR academic setting. The hands-on experience provides a valuable training approach, with more opportunities for the rehearsal of high-acuity, low-occurrence (HALO) procedures performed in IR.

## Introduction

Patient-specific three-dimensional (3D) printing is considered to be a high-growth area of medical education as digital technology advances, providing more hands-on experiences for students and clinicians to study point-of-care treatment and interventional procedures [1]. As compared to the use of cadavers, 3D printing provides countless opportunities to reproduce and scale the anatomy, as well as rehearse the dissection and repair of the anatomical model prior to performing the procedure on a patient [2]. Advancements in this area benefit clinical learning for physicians and trainees, as well as patients [3]. With the rise of 3D printing, such anatomical models are readily accessible for download using open source websites for free or a nominal fee (e.g. http://www.yeggi.com) [4]. As 3D printing becomes more advanced and widely used, many medical specialties have seen the increased utilization of educational tactile tools in combination with traditional didactic teaching methods [5]. Cardiovascular surgery, for example, has successfully been using 3D printing as an adjunct training tool with medical imaging, to surgically plan complex patient procedures and chest wall resections [6].

For interventional radiology (IR), such emerging simulation-based medical education (SBME) is also thought to be an ideal learning method, providing an accurate and economical means to study human anatomy and vasculature [7]. More specifically, select anatomy and vasculature can be extracted from patient-specific, anonymized CT or MRI scans for the purpose of teaching or analyzing the physical structure [8]. Similar to cardiology, the application of 3D printing in IR is also an ideal way to optimize procedural training strategy, allowing learners to rehearse the procedure under fluoroscopy, complete with surgical landmarks [9]. Such advancements in IR hold the potential to reduce procedural operating time, thus reducing the amount of time a patient is exposed to radiation and anaesthetics [10]. Additionally, IR has adopted the utilization of 3D printing for complex surgical planning of life-threatening abdominal aortic aneurysm, also known as a Triple A (AAA) [11]. Such vasculature can be 3D printed and the procedure practiced with guide wires, catheters, and stents to simulate the actual patient procedure, to potentially anticipate or mitigate any intra-procedural complications [12].

Using a program evaluation approach, the purpose of this technical report is to describe the development and application of 3D-printed vasculature models within a radiology interest group (composed of junior level medical school trainees) to collect their thoughts about the use of such supplementary learning tools to traditional, lecture-based teaching. Participants of the interest group were taught using a combination of traditional learning methods in combination with a tactile component to better understand the anatomy and application of the knowledge. All 30 workshop participants contributed their feedback through a quantitative survey to gain information about their experience using the models as a supplementary learning tool.

This report has three components: (a) the production of the 3D-printed anatomical models, including thoracic and lower limb vasculature; (b) the utilization of the models to better understand if they improve medical student knowledge about IR procedures; (c) the evaluation of the efficacy of the models through the use of a quantitative survey.

## Technical report

**Figure 1:**
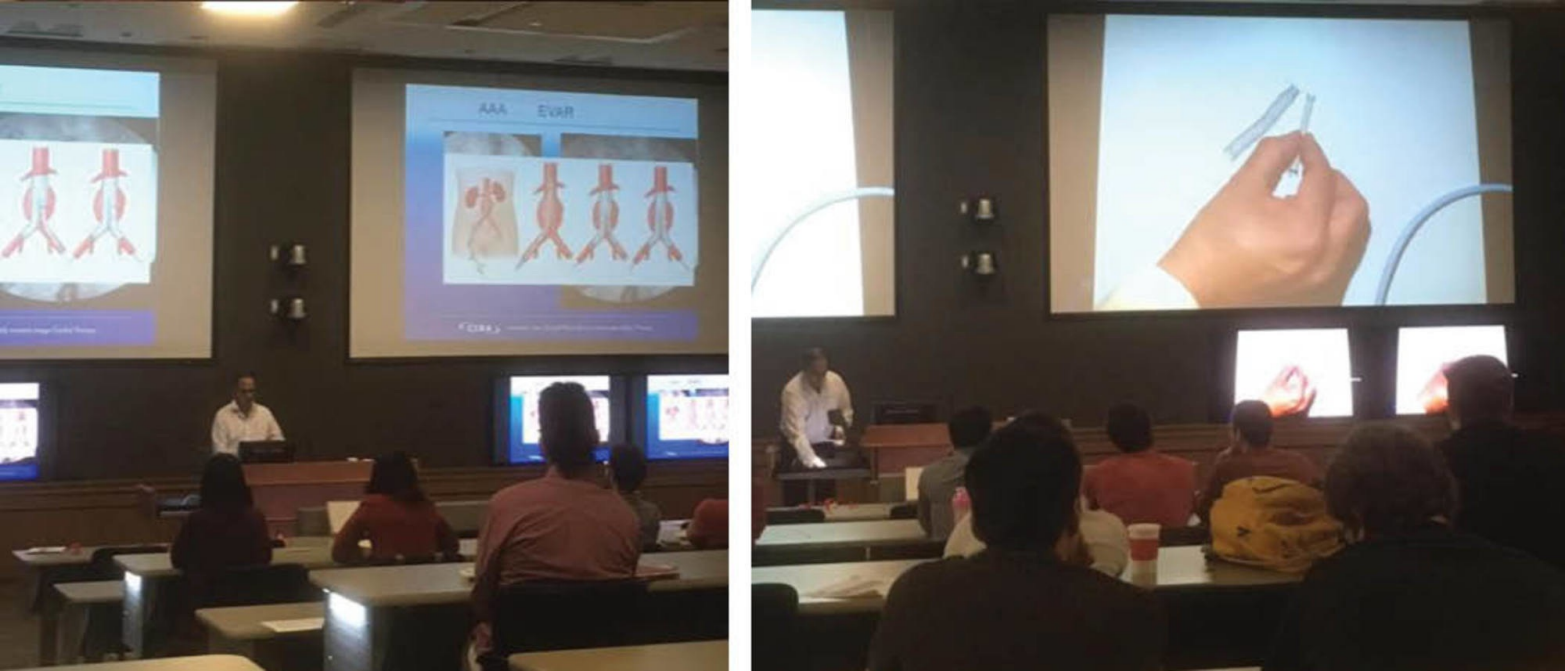
Radiology interest group training session at Memorial University of Newfoundland on March 31, 2017.

Four polymer 3D-printed vasculature models were used during a radiology interest group presentation at Memorial University of Newfoundland (MUN) on March 31, 2017, which included 30 undergraduate medical student participants of varying years in their education. The session was taught by a medical educational facilitator, (author RG - interventional radiologist, diagnostic imaging, faculty of medicine, MUN) and two medical student facilitators from MUN, (authors JK, MB). The session was one hour in duration, which included a Powerpoint presentation, demonstrating how to rehearse the insertion of guide wires and stents using 3D-printed vasculature models (Figure 1). At the end of the traditional lecture session, the interest group participants were provided an opportunity to interact with the models, as well as to attempt several procedures demonstrated during the lecture under the direct supervision by the educational facilitator.

Input

The 3D printers used to produce the models included an Ultimaker 2 (Ultimaker, Geldermalsen, Netherlands) and a Lulzbot Taz^TM^ (Aleph Objects, Inc., Loveland, Colorado, US) each with the capability to print using rigid polylactic acid (PLA) filament as well as semi-flexible NinjaTek Cheetah^TM^ (Fenner Drives, Inc., Hessle, UK) filament. Each filament material was considered to have both pros and cons associated with the printing of the vasculature. The PLA, although economical ($24/1kg - www.shop3d.ca) is very rigid and does not provide a realistic simulation experience when attempting to replicate the tissue of arterial walls. The NinjaTek Cheetah is more expensive ($120/1kg - www.thor3d.ca) but provides a more realistic representation of such soft tissue texture. The 3D-printed vasculature models created for the study were limited in size due to the dimensions of the print beds, measuring 9” x 10” and 11.75" x 11. 75”, respectively.

Process

The 3D-printed models used during the session were extracted and printed from anonymized CT and MRI scans, as well as open source online libraries such as Osirix Lite Library (www.osirix-viewer.com). The first 3D-printed vasculature model included an abdominal aorta, extracted from an anonymized CT scan and printed on an Ultimaker^ ^2 3D printer, using PLA in tandem with polyvinyl alcohol (PVA) dissolvable support material. The prints were soaked to remove the PVA and dried to ensure luminal patency. The initial prints were produced smaller (one-third) compared to the actual file size in an attempt to test the material and to also accommodate the dimensions of the print bed. Once printed, the rigid PLA was determined to be economical in cost (approximately less than $10 CAD) but unsuitable to simulate the arterial wall structure of the vasculature for simulation purposes. For this reason, the models were meant primarily for guide wire and stent insertion and positioning but not accurate for assessing the risk of arterial wall perforation.

**Figure 2:**
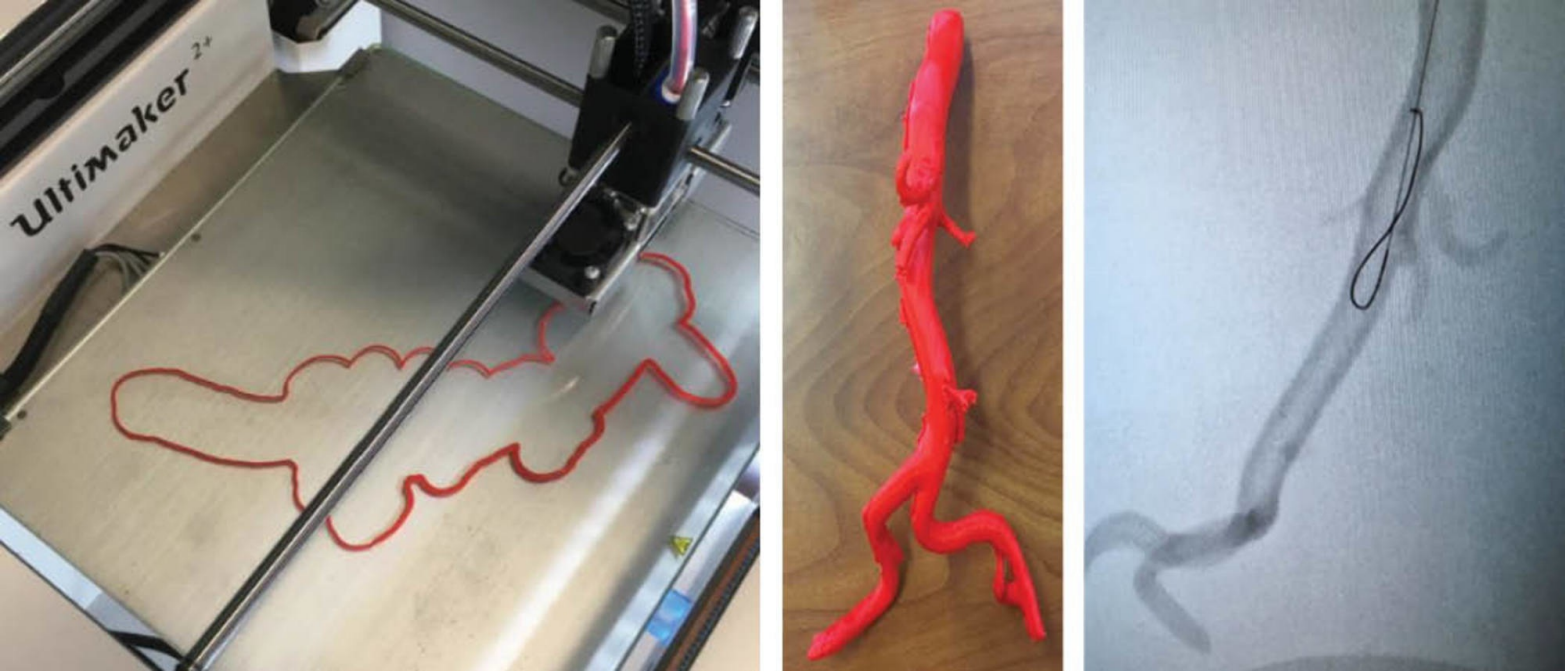
Vasculature models produced using PLA on an Ultimaker 2 3D printer

Fluoroscopy has been used as an imaging modality to test similar 3D printed models in the simulation realm and allows an opportunity to rehearse procedures using the medical-grade devices and implants in a safe, zero-risk environment [13]. The same method was used by the educational facilitator to determine if the aesthetically satisfactory models produced were effective as a simulation training tool. The models tested appeared to be functional as an anatomically realistic simulation tool with the exception of several incomplete arterial branches that were filled while printing (Figure 2). 

**Figure 3:**
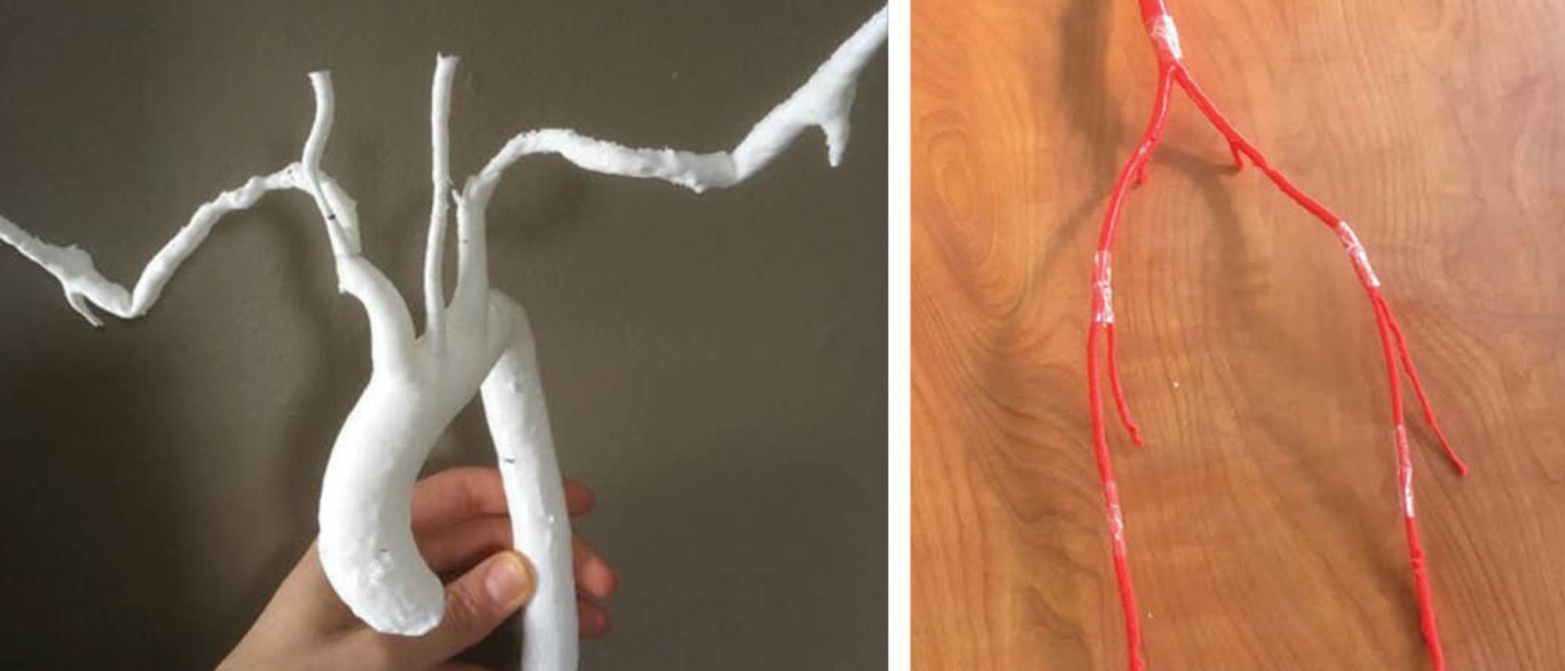
Vasculature models produced using PLA on a Lulzbot Taz 3D printer

The second 3D-printed model was extracted from an open-source CT scan of a thoracic aorta to create an aortic arch. This model was printed with white PLA on a Lulzbot Taz 3D printer. The third 3D-printed model included extracted vasculature from a CT peripheral angiogram and was printed using red PLA. This print was created in segments and later joined with tape to create a life-size adult model without reduced scaling (Figure 3).

**Figure 4:**
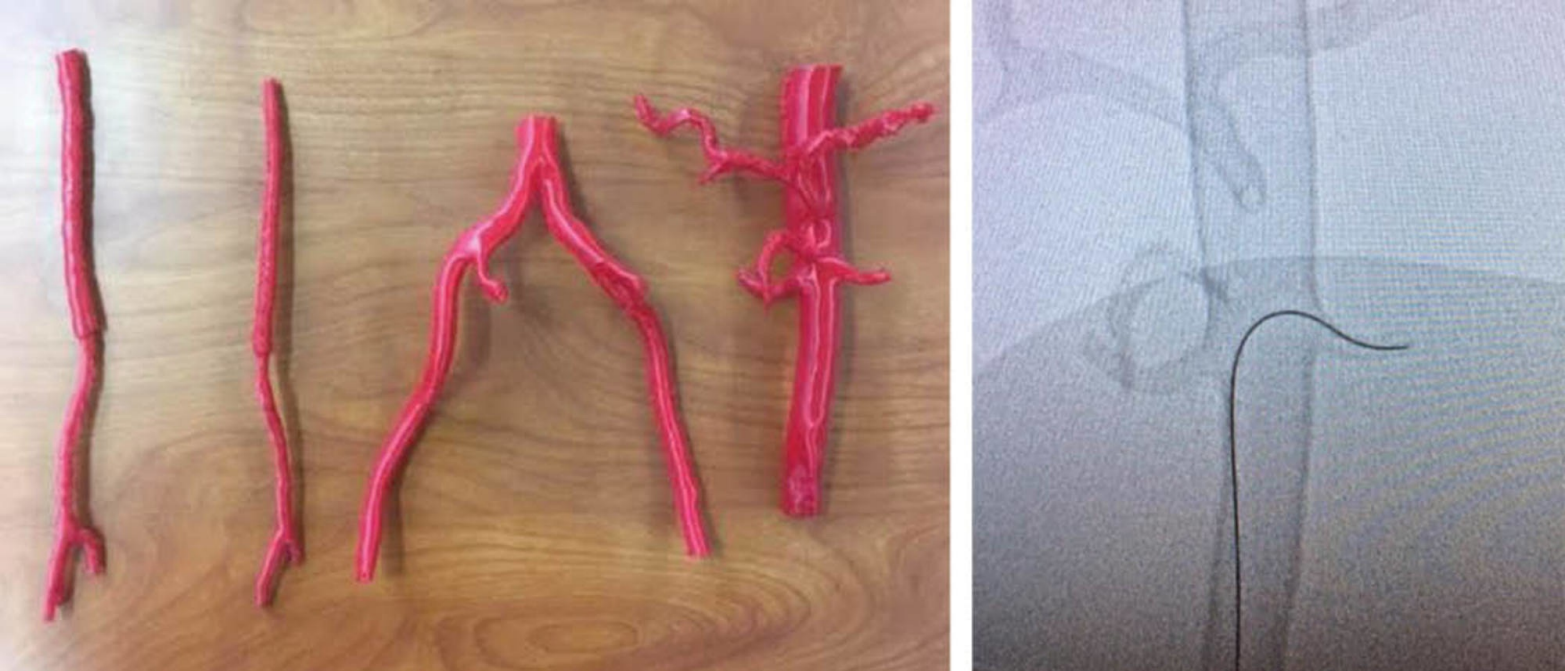
Vasculature models produced using NinjaFlex on a Lulzbot Taz 3D printer

For the fourth 3D-printed model, an abdominal aorta containing iliac branches was derived from an open source MRI. A semi-flexible NinjaTek Cheetah polymer filament was used for this print, on a Lulzbot Taz 3D printer. The NinjaTek Cheetah material was considered by the medical student facilitators to be a more ideal option compared to the PLA in terms of increased realism simulating the texture of a human artery (Figure 4). 

The difficulty encountered with NinjaTek Cheetah material was its tendency to fill in the smallest arterial branches causing difficulties in simulating accurate luminal patency. The models were viewed under fluoroscopy for testing purposes and to identify which arterial branches were obstructed from the 3D printing process. 

Products

**Figure 5:**
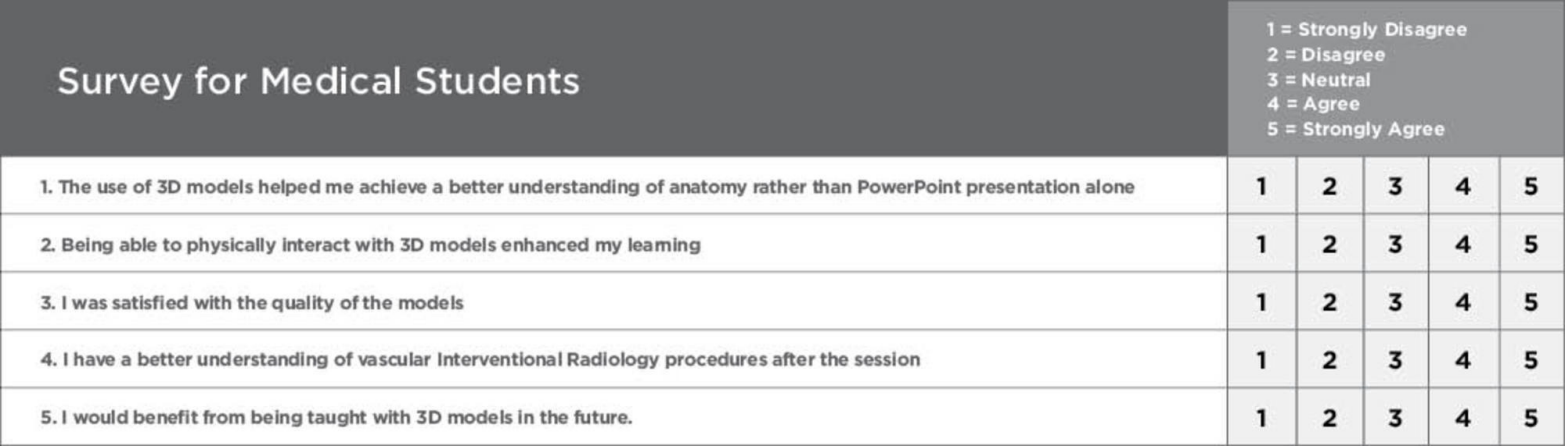
Quantitative survey for students

Following the radiology interest group Powerpoint presentation, participants examined the physical properties and provided written feedback to the facilitators. The two medical student facilitators distributed a survey to determine the efficacy of the vasculature models as supplementary training tools, compared to traditional learning methods (Figure 5). 

**Figure 6:**
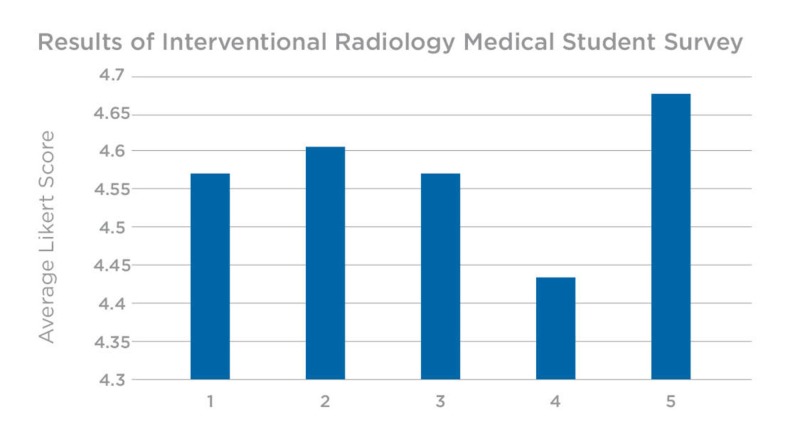
Survey results from radiology interest group at MUN x axis: survey question #, y axis: question score, MUN - Memorial University of Newfoundland

The survey was completed by all 30 undergraduate medical students to evaluate the efficacy of the 3D-printed models as an adjunct to the traditional, didactic teaching within IR. Overall, the evaluations were very positive with small standard variations. More specifically, the highest scored survey question (#5) was related to whether or not the students felt that such models and hands-on practice should be added as a learning modality to augment traditional, lecture-based teaching in the future. This question also contained the least amount of variation in response. The lowest scored question (#4) involved their overall knowledge and understanding of IR in general. However, this score was still high (4.43 out of 5), supporting the use of 3D-printed models in IR education (Figure 6).

## Discussion

The use of 3D models provided a superior learning experience in comparison to traditional learning methods. The hands-on experience provides a more simulation-based approach, which affords more opportunities for the rehearsal of high-acuity, low-occurrence (HALO) procedures as those performed in IR.

If implemented within future interest group teaching sessions, a facilitator could expect to pay $75-$100 solely for the filament material required to print four similar models. This would be a cost-recovery estimate and would not include 3D-modelling design time and technical staff time dedicated. The models can, however, be reused indefinitely as long as they are not used for incision rehearsal. For the purpose of inserting guide wires and catheters, the models are considered to be a long-term investment, with the potential to last years without obvious signs of wear. Based on the numbers of students who attend such lectures, there is a thought that the hands-on time is limited in order for all student participants to practice using such models. Evidence suggests that four participants constitute an ideal group size per instructor-or potentially, per set of tools for learning in this case [14]. A more effective way to integrate such models could be by providing an opportunity for students to purchase their own IR vasculature models through a local source.

**Figure 7:**
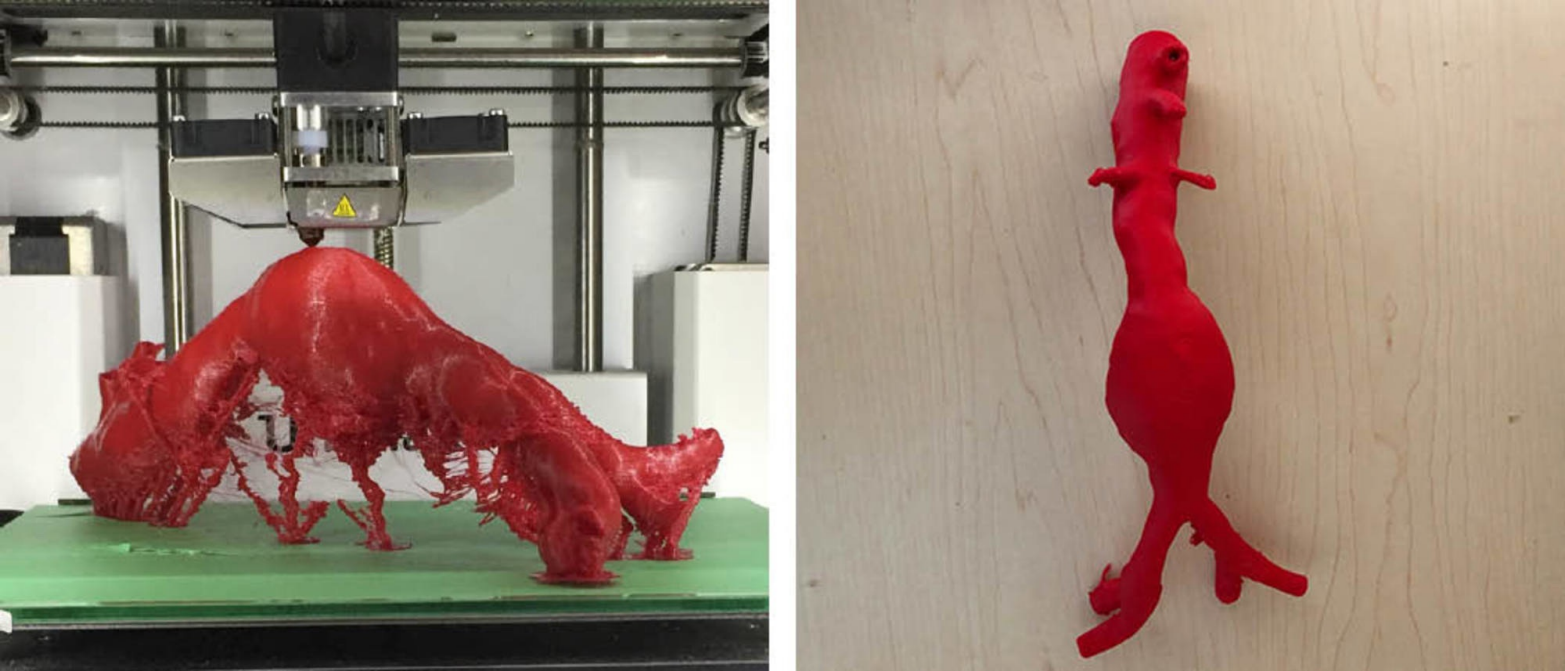
Abdominal aortic aneurysm (AAA)

MUN Med 3D has recently proposed an interventional radiology kit designed for medical students and residents which would include such vasculature for the purpose of individual training. The IR kit would include the thoracic artery, abdominal aorta, iliac branches-modular in nature so they can be assembled to create an adult-sized piece of vasculature for the rehearsal of IR procedures. Ideally, the kit will also include vasculature with common pathologies as seen in IR, such as an abdominal aortic aneurysm (Figure 7). 

Such kits could be commercially available to students at a reduced cost (estimated $250) based on the lower overhead cost of producing such products within a university environment as compared to currently available commercial alternatives, which can cost upwards of $2000 [15].

**Figure 8:**
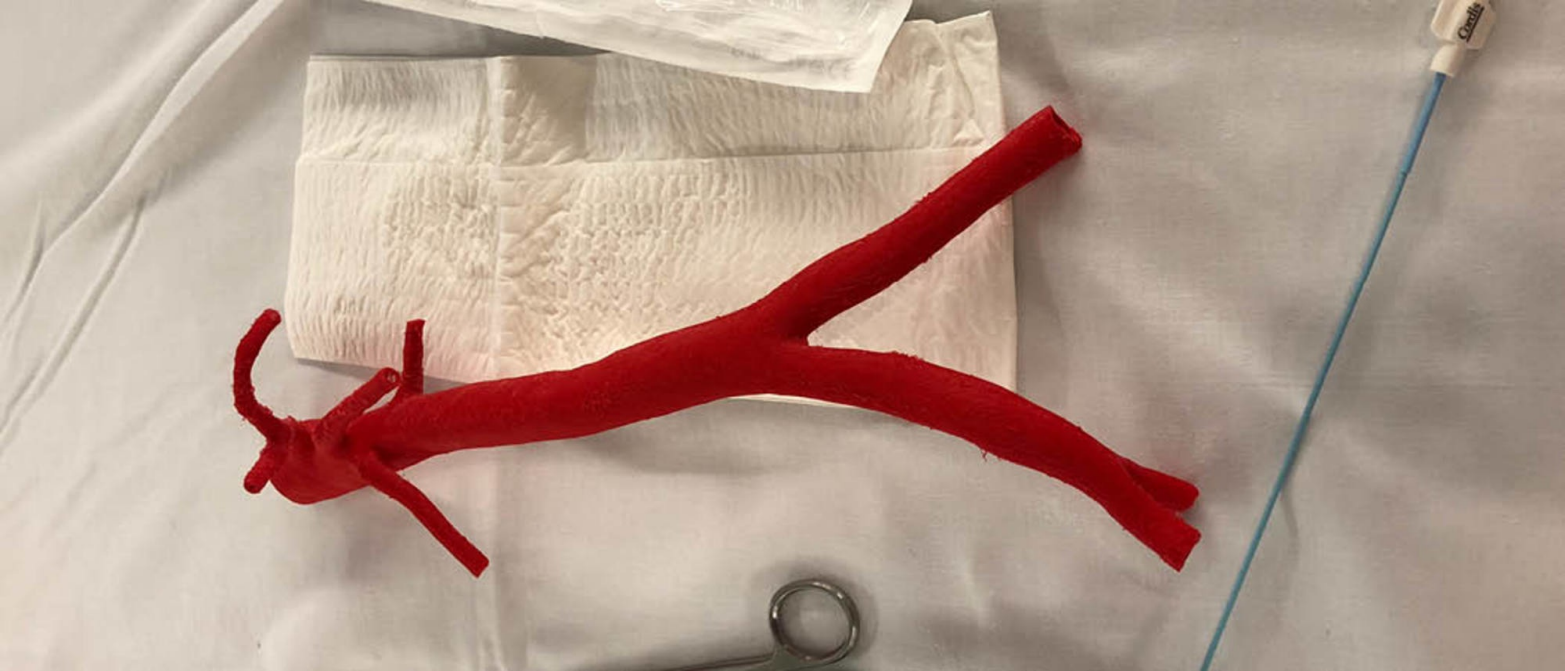
3D-printed abdominal aorta using TPE 80A filament

Several prototype modifications are to be considered prior to future models for learning or the production of such an IR Kit: (a) print vasculature using an alternative material called Thermoplastic elastomer (TPE 80A) ($52/1kg - www.filaments.ca) which prints higher quality vasculature with improved luminal patency, (b) attempt to clear all vasculature branches during post-production soaking to remove PVA, (c) 3D-print vasculature with and without pathologies to teach the anatomic properties of each. Prior to this technical report, a follow-up 3D print was produced from a de-identified CT scan of an abdominal aorta. The TPE 80A printed without as much interior infill and its branches were less obstructed than prints using the NinjaTek Cheetah^TM^ filament. The added benefits of the TPE material is that it prints with a flexible, smooth and rubbery finish compared to alternative filament materials (Figure 8).

**Figure 9:**
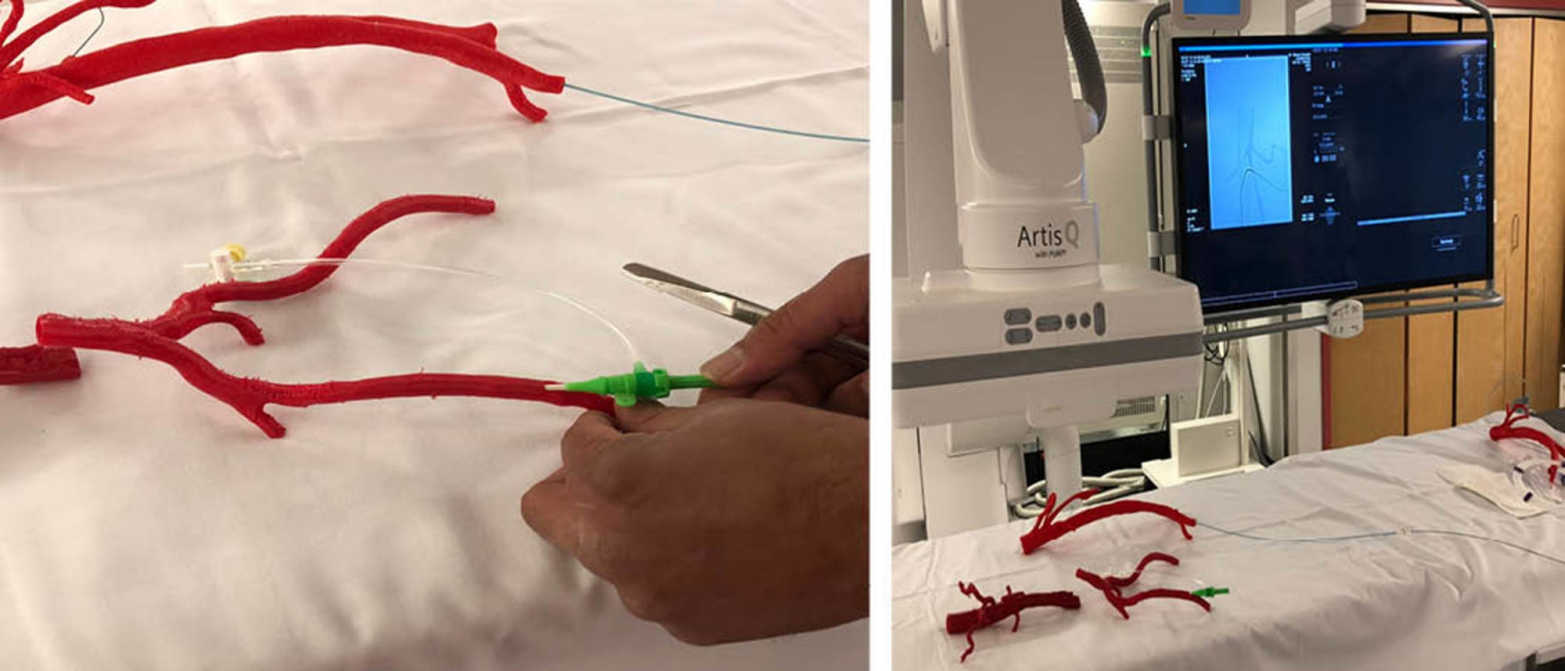
Two 3D-printed models tested under fluoroscopy.

To ensure the proposed material was more accurate, an abdominal aorta was produced by MUN Med 3D, and tested by the same educational facilitator on July 13th, 2018. Under fluoroscopy, the femoral artery of the latest model was used as an access point to insert a guide wire. The model was helpful to better understand which improvements are required when using the TPE 80A material. The educational facilitator determined that the smaller branches were filled but could still be functional if cut towards the anterior edge of each branch (Figure 9).

## Conclusions

This technical report described the development of 3D-printed models for simulation purposes within IR, and the evaluation of adding this component to traditional, didactic teaching. The results of these evaluations reveal that 3D-printed models are a cost-effective and advanced means to teach procedural skills more effectively when they serve as supplementary tools to traditional learning methods. The 3D-printed models were evaluated by the IR student interest group participants to be beneficial with respect to the hands-on spatial perception and tactile feedback. The highest scored question on the quantitative survey pointed to the desire of students to have such models as part of their ongoing SBME. This study was a positive indicator that such models can potentially benefit outcomes for students and residents learning interventional radiology.
